# Characterizing neurodevelopmental follow-up attendance of children with congenital heart disease

**DOI:** 10.1038/s41390-025-04247-y

**Published:** 2025-07-15

**Authors:** Gautam Dagur, Jake A. Kleinmahon, Michelle Z. Gurvitz, Rebeka Acosta, Samudragupta Bora

**Affiliations:** 1 Mater Research Institute, Faculty of Health, Medicine and Behavioural Sciences, The University of Queensland, Brisbane, QLD Australia; 2 Ochsner Clinical School, Faculty of Health, Medicine and Behavioural Sciences, The University of Queensland, New Orleans, LA USA; 3 Division of Pediatric Cardiology, Cohen Children’s Medical Center, Queens, NY USA; 4 Department of Pediatrics, Zucker School of Medicine at Hofstra/Northwell, New Hyde Park, NY USA; 5 Department of Pediatrics, Harvard Medical School, Boston, MA USA; 6 Department of Cardiology, Boston Children’s Hospital, Boston, MA USA; 7 Conquering CHD, Madison, WI USA; 8 A+J Patient Advocacy, LLC, Las Vegas, NV USA; 9 Health Services Research Center, University Hospitals Research & Education Institute; Department of Pediatrics, University Hospitals Rainbow Babies & Children’s Hospital, Case Western Reserve University School of Medicine, Cleveland, OH USA

## Abstract

**Background:**

Characterize attendance at neurodevelopmental follow-up for congenital heart disease (CHD) from infancy to young adulthood, as well as identify individual, family, and neighborhood-level correlates of low attendance.

**Methods:**

Primary caregivers (*N* = 578) of children with CHD aged 0–21 years in the United States, stratified into six age groups, reported whether their child had attended neurodevelopmental follow-up at various time points. Caregiver reports were also used to collect information on the child’s clinical and family psychosocial characteristics. ZIP codes provided neighborhood-level information.

**Results:**

Overall, 51% (*n* = 296/578) of children with CHD had attended at least one age-appropriate neurodevelopmental follow-up. Attendance relative to age-appropriate appointments decreased with age, with <3% aged 6–21 years attending 100% of age-appropriate follow-up. Low (<50% age-appropriate) attendance was associated (*p* < 0.05) with older child age, racial and ethnic minority group status of mother, more social support from extended family, shorter duration of first ICU stay, lack of grandparents’ involvement in childcare, travel distance of ≥60 miles from pediatric cardiology center, limited economic and educational opportunities in the neighborhood, and biventricular diagnosis.

**Conclusions:**

The findings underscore the necessity of targeted intervention, with a particular emphasis on the social determinants of health framework, to improve the attendance of children with CHD at neurodevelopmental follow-up.

**Impact:**

- In a U.S. sample, primary caregivers reported that 51% of children with congenital heart disease attended at least one age-appropriate neurodevelopmental follow-up appointment.
- Attendance rates declined with age, and clinical factors like shorter initial ICU stay and biventricular diagnosis were associated with low attendance.
- Low attendance was also associated with non-clinical factors like maternal race and ethnicity, family social support, proximity to the cardiology center, and economic and educational opportunities in the neighborhood, highlighting the importance of taking social determinants of health into account while considering opportunities to increase neurodevelopmental follow-up attendance.

As highlighted in the American Heart Association (AHA) and American Academy of Pediatrics (AAP) scientific statement on neurodevelopmental outcomes, congenital heart disease (CHD) has adverse impacts on the quality of life and life-course opportunities of a considerable number of children.^1^ Recognizing neurodevelopmental delays/deficits early is important for the timely detection of impairments and targeted interventions to improve outcomes of high-risk populations. Consequently, attending neurodevelopmental follow-up appointments at key developmental milestones is critical.

A wide variability (11–76%) in attendance at cardiology follow-up has been documented among the CHD population, with loss to follow-up associated with low family socioeconomic status (SES), minority race and ethnicity, referral from a non-cardiology specialist, and lack of awareness among patients and parents regarding the availability and importance of follow-up care.^2–5^ A recent systematic review identified additional factors for loss to follow-up, such as public health insurance/self-payer status, limited transportation access, low parental educational attainment, and immigrant and refugee status.^6^ Thus, it is clear that social determinants of health and disparities in healthcare access have a significant impact on the successful follow-up of children with CHD.

Despite the importance of neurodevelopmental follow-up in children with CHD, little is known about its uptake among patients and families. Three recent retrospective studies from the United States (U.S.) have shed some light on this issue. Across two studies, rates of neurodevelopmental follow-up were lower than cardiology follow-up.^7,8^ In the single-center study at the University of Utah, only 4% of at-risk children with CHD attended follow-up appointments during standard evaluation ages (1–2 years, 4–6 years, and 8–14 years) between June 2015 and December 2017.^7^ A low attendance rate was associated with a lack of referring pediatric cardiologists in the area, and no referrals were made by primary care physicians or family/self-referrals.^7^ Among those with a neurodevelopmental follow-up referral, 30% were no-shows or canceled within 24 hours of the appointment.^7^ Another single-center study at the University of Michigan C.S. Mott Children’s Hospital found that only 17% of 552 children who underwent cardiac surgery under the age of 1 year between April 2011 and March 2014 completed any neurodevelopmental assessment at the specified evaluation ages (9–12 months, 18–24 months, and 3 years).^8^ Non-attendance was associated with not having an established cardiologist at the center, living further away from the surgical center, parents who were not college graduates, and low family SES including families with low income and lack of private insurance.^8^

In the third retrospective study, Michael et al. implemented an initial consultation protocol to educate families about the potential risks of neurodevelopmental delay and the importance of neurodevelopmental surveillance in high-risk children with CHD after cardiac surgery.^9^ To assess the protocol’s effectiveness, 85 children were recruited between October 2013 and October 2014, and 38 children between July 2012 and December 2012, both before and after the protocol was implemented.^9^ Attendance at the initial neurodevelopmental surveillance was 21% in the pre-protocol period and 82% in the post-protocol period, with a higher retention rate for subsequent follow-up appointments—6 months for routine and 3 months for those with neurological concern—of 38% in the pre-protocol and 52% in the post-protocol period.^9^

Although these studies identify several factors associated with attendance at neurodevelopmental follow-up for CHD, there are still numerous clinical and methodological issues that require further investigation and clarification. The first concern pertains to the single-center design, particularly restricted to large academic medical centers. Hence, the generalizability of their findings to the broader population of CHD, even across the U.S., remains unclear. A related issue concerns the limited assessment time points, with little to no information across major developmental transitions and longitudinal patterns of attendance at follow-up. Finally, existing studies are limited by a narrow conceptualization of the predictors of attendance without adequate consideration for the social determinants of health framework, despite prevailing disparities in access to healthcare among this high-risk population.^6^ Against this background, drawing on data from a cross-sectional survey in the U.S., the aims of this study were as follows.

1. To characterize the patterns of attendance at neurodevelopmental follow-up of CHD from infancy to young adulthood.
2. To identify individual, family, and neighborhood factors associated with low (<50% age-appropriate) attendance at neurodevelopmental follow-up of CHD.

## Methods

### Participants

Primary caregivers of children with CHD were recruited online in the U.S. between November 2020 and January 2021 using social media (e.g., Facebook, Twitter) and patient and family advocacy organizations (e.g., Conquering CHD). The following criteria were used to determine inclusion: 1) the respondent was the primary caregiver of a child with CHD between the ages of 0 and 21 years; 2) the respondent’s age ranged from 22 to 64 years; and 3) the respondent’s primary language (not necessarily native) was English. Furthermore, the respondent’s report of the child’s CHD diagnosis must be consistent throughout the screening and outcomes assessment, and they must respond with “confident,” “very confident,” or “extremely confident” on a five-point Likert scale of confidence. Respondents who were primary caregivers of more than one child with CHD were asked to refer to their youngest child with CHD for this study.

**Fig. 1:**
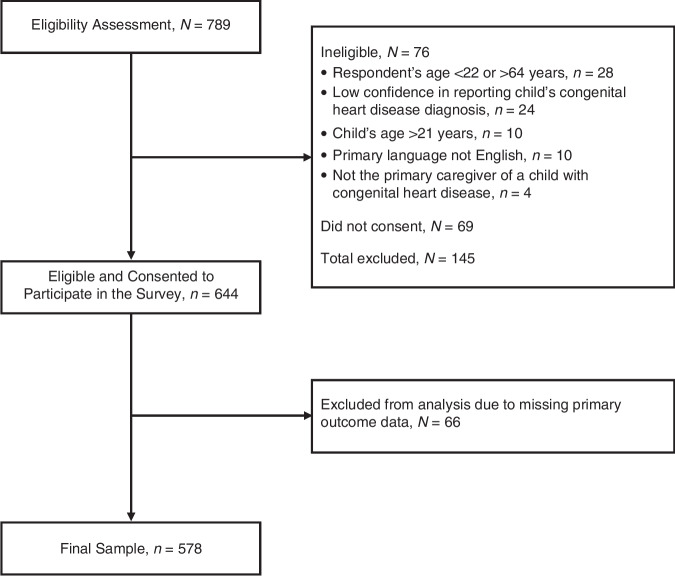
Participant recruitment flowchart. This figure depicts the recruitment procedure for the study sample, as well as the total number of those excluded and their reasons.

As shown in Fig. 1, 789 primary caregivers were evaluated for eligibility; 145 (18%) were excluded because they did not meet the inclusion criteria or did not consent to participate in the study, and 66 (10%) were excluded due to missing outcomes data, leaving 578 (73%) primary caregivers eligible to participate in the current study. Based on the child’s age, the sample was stratified into six age groups: <1 year (*N* = 55), 1–<3 years (*N* = 143), 3–5 years (*N* = 115), 6–9 years (*N* = 104), 10–17 years (*N* = 123), and 18–21 years (*N* = 38).

The survey adhered to the American Association for Public Opinion Research’s Best Practices for Survey Research. Survey items were developed in collaboration with experts in survey methodology, high-risk infant follow-up including CHD, professional organizations of parents with CHD, and, most importantly, individuals with lived experiences (i.e., primary caregivers of children with CHD) to ensure clarity and relevance. Pilot testing was carried out with key stakeholders, including cognitive testing of survey items. All study procedures were reviewed and approved by the University of Queensland Medicine, Low & Negligible Risk Ethics Sub-Committee (Approval #2018001331). All participants provided electronically documented informed consent to participate in the study. This study adhered to the Strengthening the Reporting of Observational Studies in Epidemiology reporting guidelines for cross-sectional studies.

### Measures

The survey was administered via a secure web-based portal. The primary outcome of interest was primary caregiver-reported attendance of their child at clinical neurodevelopmental follow-up appointments for CHD at various time points. Clinical neurodevelopmental follow-up was defined in this study as monitoring and assessing child developmental outcomes in cognition, language, behavior/mental health, physical health, and motor, among others, as part of clinical care rather than a research study. Age-appropriate appointments were determined using the child’s current age, with each age group corresponding to the completion of one appointment. The total number of age-appropriate appointments for each age group is as follows: <1 year (1 appointment), 1–<3 years (2 appointments), 3–5 years (3 appointments), 6–9 years (4 appointments), 10–17 years (5 appointments), and 18–21 years (6 appointments). These age groups were informed by the 2012 Scientific Statement of the American Heart Association, which is also consistent with the most recent 2024 update to the statement.

Primary caregiver responses provided information on child clinical, family psychosocial, and neighborhood characteristics (see Table 1). The primary caregiver-reported CHD diagnosis was further reviewed by the study team’s pediatric cardiologist for reported and missing data for single ventricular diagnosis. Two additional variables were identified based on primary caregiver-reported information: grandparents’ and extended family members’ involvement in childcare with primary caregivers. Grandparent support included grandmother, grandfather, stepparent’s mother or father, or fiancé's parent; extended family support included the involvement of an uncle, aunt, or cousin.

**Table 1:** Child, mother, and family characteristics of the sample.

Characteristics, % (*n*/*N*)	Infancy [<1 year]	Toddlerhood [1–<3 years]	Early childhood [3–5 years]	Late childhood [6–9 years]	Adolescence [10–17 years]	Young adulthood [18–21 years]	*p*
	*N* = 55	*N* = 143	*N* = 115	*N* = 104	*N* = 123	*N* = 38	
*Child*							
Male sex	53 (29/55)	59 (84/143)	59 (68/115)	53 (55/104)	63 (77/123)	53 (20/38)	0.65
Preterm birth [<37 weeks gestation]	26 (14/54)	19 (27/142)	16 (18/114)	20 (21/104)	17 (21/121)	16 (6/38)	0.70
Antenatal congenital heart disease diagnosis	71 (39/55)	57 (81/143)	59 (67/114)	44 (46/104)	39 (47/121)	29 (11/38)	<0.001
Single ventricle physiology	27 (15/55)	25 (36/143)	35 (40/115)	25 (26/104)	32 (39/123)	32 (12/38)	0.51
Open heart surgery	82 (45/55)	90 (128/143)	90 (103/115)	89 (92/103)	89 (109/123)	92 (35/38)	0.66
Heart transplant	0 (0/54)	2 (3/143)	3 (3/115)	2 (2/104)	2 (3/123)	5 (2/38)	0.73
Extracorporeal membrane oxygenation	21 (11/53)	29 (38/130)	24 (25/105)	24 (22/93)	28 (29/102)	27 (9/33)	0.80
Extended first intensive care unit stay [>21 days]	31 (17/55)	46 (64/138)	44 (49/111)	39 (41/104)	35 (43/122)	27 (10/37)	0.12
*Mother [at childbirth]*							
Young motherhood [<25 years]	12 (6/49)	10 (13/137)	15 (16/106)	11 (11/100)	21 (24/116)	20 (7/35)	0.13
Single motherhood	7 (4/54)	5 (7/142)	12 (13/113)	3 (3/102)	5 (6/118)	5 (2/37)	0.14
Low academic attainment	9 (5/55)	13 (18/140)	6 (7/113)	5 (5/102)	9 (11/118)	11 (4/37)	0.31
Racial and ethnic minority groups	17 (9/52)	11 (16/141)	10 (11/113)	8 (8/103)	5 (6/119)	5 (2/37)	0.14
Racial and ethnic group							
American Indian or Alaska Native	0 (0/52)	0 (0/141)	1 (1/113)	0 (0/103)	0 (0/119)	0 (0/37)	
Asian	4 (2/52)	2 (3/141)	1 (1/113)	2 (2/103)	2 (2/119)	0 (0/37)	
Black or African American	4 (2/52)	1 (1/141)	0 (0/113)	1 (1/103)	2 (2/119)	0 (0/37)	
Hispanic or Latino	10 (5/52)	8 (11/141)	8 (9/113)	4 (4/103)	0 (0/119)	5 (2/37)	
Native Hawaiian and Pacific Islander	0 (0/52)	0 (0/141)	0 (0/113)	0 (0/103)	1 (1/119)	0 (0/37)	
White or Caucasian	83 (43/52)	89 (125/141)	90 (102/113)	92 (95/103)	95 (113/119)	95 (35/37)	
Other	0 (0/52)	1 (1/141)	0 (0/113)	1 (1/103)	1 (1/119)	0 (0/37)	0.46
*Family [concurrent]*							
Public health insurance	38 (21/55)	31 (44/143)	28 (32/115)	22 (23/104)	20 (25/123)	26 (10/38)	0.12
Low socioeconomic status [<US$30,000 per year]	29 (16/55)	24 (34/142)	23 (27/115)	13 (14/104)	25 (31/123)	29 (11/38)	0.18
Proximity to pediatric cardiology center [≥60 miles from home]	27 (15/55)	31 (45/143)	25 (29/115)	30 (31/104)	30 (37/123)	39 (15/38)	0.67
Primary caregiver with extended family support in childcare	28 (15/53)	23 (33/142)	25 (28/113)	22 (23/103)	24 (29/122)	18 (7/38)	0.92
Primary caregiver with grandparent support in childcare	40 (21/53)	38 (54/142)	48 (54/113)	48 (49/103)	43 (53/122)	32 (12/38)	0.35
Neighborhood livability, mean ± SD							
Housing	53 ± 14	54 ± 14	53 ± 14	54 ± 14	52 ± 14	59 ± 14	0.21
Transportation	55 ± 14	51 ± 14	55 ± 13	52 ± 12	52 ± 12	55 ± 14	0.17
Environment	53 ± 15	51 ± 14	53 ± 14	54 ± 14	52 ± 15	53 ± 14	0.89
Engagement	54 ± 13	54 ± 15	54 ± 15	57 ± 15	57 ± 13	52 ± 14	0.14
Opportunity	53 ± 14	56 ± 13	55 ± 16	59 ± 13	56 ± 14	55 ± 16	0.16
U.S. geographical region of residence							
Northeast	11 (6/55)	17 (25/143)	16 (18/115)	10 (10/104)	12 (15/123)	21 (8/38)	
Midwest	36 (20/55)	30 (43/143)	30 (35/115)	38 (40/104)	38 (47/123)	37 (14/38)	
South	27 (15/55)	31 (44/143)	27 (31/115)	33 (34/104)	33 (40/123)	34 (13/38)	
West	25 (14/55)	22 (31/143)	27 (31/115)	19 (20/104)	17 (21/123)	8 (3/38)	0.44

Additional data were extracted using primary caregiver-reported ZIP code: 1) proximity to a pediatric cardiology center, as part of a free-standing children’s hospital or a larger hospital/healthcare organization with or without academic affiliation; 2) neighborhood quality metrics; and 3) geographic region of residence in the U.S. The distance to the nearest pediatric cardiology center was calculated by drawing a straight line from the home ZIP code to the pediatric cardiology center’s ZIP code using the U.S. News and World Report ranking list of pediatric cardiology and heart centers. Additional centers were identified for states that did not have a hospital on the list. These centers were cross-matched based on primary caregiver-reported follow-up locations and travel distance.

### Statistical analyses

Data were analyzed using IBM^®^ SPSS^®^ software version 27.0. Sample characteristics and outcomes for categorical variables are expressed as a percentage (numerator/denominator), and continuous variables are expressed as a mean with standard deviation. The Chi-square test of independence for categorical variables and analysis of variance for continuous variables were used to analyze differences between child age groups. Statistical significance was set at *p* < 0.05.

Multivariable logistic regression was used to identify factors independently associated with low attendance at neurodevelopmental follow-up (defined as completing <50% of age-appropriate appointments). The regression used three models: individual, family, and neighborhood, based on the social determinants of health framework.^10–12^ The variables used in each model were 1) individual (child): sex, age, ventricular classification, antenatal CHD diagnosis, open heart surgery, first intensive care unit (ICU) stay duration; 2) family: low SES (<US$30,000 per year), multigenerational support in childcare, extended family support in childcare, insurance status, maternal academic underachievement, young motherhood, racial and ethnic minority group status of mother; 3) neighborhood: moderate-to-low housing, transportation, environment, engagement, and opportunity metrics; patient and family advocacy chapter in the state of residence, and proximity to a pediatric cardiology center.

## Results

The current sample of children, drawn from 578 responses by primary caregivers, included 58% males and 29% with a single ventricle diagnosis. Antenatal CHD diagnosis varied significantly across the child age groups. Table 1 summarizes the sample’s child clinical, family psychosocial, and neighborhood characteristics.

### Neurodevelopmental follow-up attendance

**Fig. 2:**
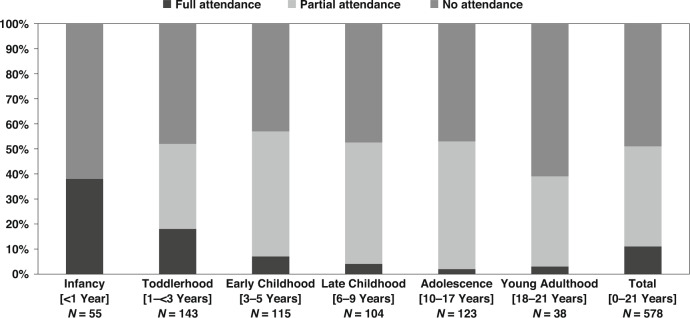
Neurodevelopmental follow-up attendance of children with congenital heart disease^a^. This figure depicts the attendance patterns for children with congenital heart disease at neurodevelopmental follow-up from infancy to young adulthood. ^a^Full attendance = 100%; Partial attendance = >0% and <100%; No attendance = 0% of total age-appropriate appointments. During infancy [<1 year], no partial attendance as the maximum eligible appointment is one.

As shown in Fig. 2, 51% (*n* = 296/578) of primary caregivers reported their child had attended at least one age-appropriate neurodevelopmental follow-up appointment for CHD. Nonetheless, attendance relative to age-appropriate appointments decreased with age, with <3% aged 6–21 years attending 100% of eligible appointments. Furthermore, as shown in Table 2, there was a decline in attendance rates following toddlerhood, with ≤20% of children with CHD attending neurodevelopmental follow-up appointments during early childhood and later.

**Table 2:** Neurodevelopmental follow-up attendance at different time points among children with congenital heart disease.

Follow-up time points, % (*n*/*N*)^a^	Child age						Follow-up attendance, % (*n*/*N*) eligible per time point
	Infancy [<1 year] *N* = 55	Toddlerhood [1–<3 years] *N* = 143	Early childhood [3–5 years] *N* = 115	Late childhood [6–9 years] *N* = 104	Adolescence [10–17 years] *N* = 123	Young adulthood [18–21 years] *N* = 38	
Infancy [<1 year]	38 (21/55)	37 (53/143)	36 (41/115)	20 (21/104)	20 (25/123)	11 (4/38)	29 (165/578)
Toddlerhood [1–<3 years]	–	33 (47/143)	34 (39/115)	21 (22/104)	17 (21/123)	13 (5/38)	26 (134/523)
Early childhood [3–5 years]	–	–	17 (19/115)	21 (22/104)	12 (15/123)	5 (2/38)	15 (58/380)
Late childhood [6–9 years]	–	–	–	25 (26/104)	20 (25/123)	8 (3/38)	20 (54/265)
Adolescence [10–17 years]	–	–	–	–	15 (19/123)	24 (9/38)	17 (28/161)
Young adulthood [18–21 years]	–	–	–	–	–	5 (2/38)	5 (2/38)

^a^Each cell is independent and represents the number of children that attended a follow-up appointment for the age groups provided. The number of children in each age group at different follow-up time points provides cross-sectional rather than longitudinal data. Dashes in various cells indicate that a follow-up time point was not appropriate for the child’s age group.

### Correlates of neurodevelopmental follow-up attendance

As shown in Table 3, after adjusting for significant covariates, low attendance (<50% age-appropriate appointments) at clinical neurodevelopmental follow-up was associated with older (≥3 years) children (odds ratio: 7.15 [95% confidence interval: 4.60–11.12]), shorter (≤21 days) initial ICU stay (1.93 [1.26–2.97]), biventricular diagnosis (1.71 [1.08–2.70]), racial and ethnic minority group status of the mother (2.26 [1.03–4.96]), primary caregivers without grandparent involvement in childcare (1.87 [1.14–3.07]), primary caregivers with extended family involvement in childcare (2.04 [1.14–3.70]), moderate to low neighborhood opportunity (1.79 [1.11–2.87]), and greater ≥60 miles travel distance to pediatric cardiology center for follow-up services (1.79 [1.10–2.91]).

**Table 3:** Correlates of low neurodevelopmental follow-up attendance among children with congenital heart disease.

Correlates	Adjusted odds ratio (95% confidence interval)	*p*
*Individual*		
Child’s current age		
0–<3 years	Reference	
3–21 years	7.15 (4.60–11.12)	<0.001
First intensive care unit stay duration		
>21 days	Reference	
≤21 days	1.93 (1.26–2.97)	0.003
Ventricular classification		
Single ventricle diagnosis	Reference	
Biventricular diagnosis	1.71 (1.08–2.70)	0.02
*Family*		
Maternal race and ethnicity		
White or Caucasian	Reference	
Racial and ethnic minority groups	2.26 (1.03–4.96)	0.04
Multigenerational involvement in childcare		
Primary caregiver with grandparent support	Reference	
Primary caregiver without grandparent support	1.87 (1.14–3.07)	0.01
Extended family involvement in childcare		
Primary caregiver with extended family support	Reference	
Primary caregiver without extended family support	0.49 (0.27–0.88)	0.02
*Neighborhood*		
Opportunity livability index		
High livability	Reference	
Moderate-to-low livability	1.79 (1.11–2.87)	0.02
Proximity to pediatric cardiology center		
<60 miles	Reference	
≥60 miles	1.79 (1.10–2.91)	0.02

## Discussion

This cross-sectional study investigated the patterns of attendance at neurodevelopmental follow-up for children with CHD in the U.S. from infancy to young adulthood, as well as the correlates of low attendance. In the current study sample, 51% of primary caregivers reported that their child had attended at least one age-appropriate neurodevelopmental appointment; however, the follow-up rate decreased with age. This was consistent with previous research, which reported that children with CHD had a low attendance rate at neurodevelopmental follow-up appointments ranging from 4% to 52%.^7–9^ In a recent multi-center retrospective study, 29% of children with CHD aged 11–30 months attended neurodevelopmental evaluations; however, additional evaluations were performed outside the specified age range or at primary cardiac care facilities outside the institution. Due to unverified attendance data, the institutional rate of attendance varied greatly, ranging from 7.8% to 54.3%.^13^ Other high-risk populations, such as infants with hypoxic-ischemic encephalopathy and bronchopulmonary dysplasia, also showed a low rate of neurodevelopmental follow-up attendance.^14–17^

In contrast to previous research, which primarily focused on children with CHD aged 0–5 years, this study was unique in that it documented a longitudinal follow-up from infancy to young adulthood. The current study found that less than a fifth of school-aged (6–21 years) children with CHD attended neurodevelopmental follow-up. These findings align with a recent cross-sectional survey of 23 centers reporting annual cardiac neurodevelopmental evaluations that accounted for 16% of visits for children over 5 years and <3% for those over 18 years.^18^

The observed low rates of attendance, as documented in this and other studies, are most likely due to a combination of factors involving healthcare systems, healthcare professionals, and parents/primary caregivers. For example, in the recent survey by Miller et al., 53% of the 23 institutions lacked routine follow-up service for children over the age of 5 years, and only seven centers reported evaluations beyond 18 years.^18^ Further, assessments of high-risk CHD children for neurodevelopmental impairments at the Clinique d’Investigation Neurocardiaque, a Canadian program established in 2013, primarily focused their assessments between birth and 42 months.^19^

The guidelines for neurodevelopmental follow-up of children with CHD were lacking until recently. In 2012, the AHA and AAP published the first scientific statement for neurodevelopmental evaluation and management.^1^ There is no doubt that the changes in early follow-up over time reflect the increased availability of follow-up opportunities in recent years, which was also demonstrated in a recent scoping review.^20^

In addition to healthcare factors, parental/primary caregiver factors, such as their perception of the child’s well-being, have a significant impact on the uptake of high-risk follow-up. Scheduling issues, multiple appointments at different times, perceptions of appropriate development, education/unawareness for follow-up, and the primary pediatrician informing that further follow-up was unnecessary have all been identified as common reasons for declining follow-up rates.^15,17,21^ A recent study identified similar concerns, emphasizing the importance of organized and accessible developmental follow-up services to reduce the burden on parents and families.^22^ Taken together, these findings have implications for the development of novel strategies and interventions aimed at addressing these concerns to increase the uptake of neurodevelopmental surveillance and evaluation of this high-risk population and optimize outcomes.

Various individual, family, and neighborhood factors were associated with low attendance at neurodevelopmental follow-up in the current study. Consistent with previous studies, this study found individual factors, such as the older age of the child, shorter ICU stay, and biventricular diagnosis, were associated with low attendance for neurodevelopmental follow-up appointments. Shorter ICU stays and biventricular diagnoses, both of which indicate lower clinical severity of the condition, are likely to be associated with a better prognosis of neurodevelopmental outcomes than those with more severe CHD, influencing attendance at neurodevelopmental follow-ups.^8,23,24^ In contrast, Michael et al. found that children with a single ventricle CHD diagnosis were less likely to attend follow-up than those with a biventricular diagnosis.^9^ Similar findings have been reported in other high-risk populations, such as preterm birth, with children at the highest clinical risk being more likely to drop out of follow-up programs.^25^

The current study identified family correlates of low neurodevelopmental follow-up attendance, such as the racial and ethnic minority group status of the mother, lack of multigenerational childcare support, and extended family support. Racial and ethnic minority groups have been well documented in the literature as being associated with poor follow-up rates.^15,26^ Agrawal et al. aimed to better understand the cardiac needs of immigrant and refugee children and found that these patients and families had difficulty in accessing care or attending follow-up appointments.^27^ This could be due to a variety of factors, including language barriers, transportation issues, financial constraints, or insurance status.^27,28^ Parents from minority communities, who have low maternal educational attainment, low SES, and no insurance, reported greater difficulty and dissatisfaction with their children’s access to services.^29^

To our knowledge, studies to date have not investigated the relationship between multigenerational and extended family support and neurodevelopmental follow-up attendance among children with CHD. These are important variables to consider, as previous research has found that multigenerational families (living in the same house), as well as families with support from grandparents (living nearby) and extended family, provided more practical and emotional support with childcare and parental guidance.^30,31^ Family support has been linked to better healthcare outcomes for children; however, the link between extended family support and childcare is still unclear.^32,33^ The current study did not consider the extent or nature of family involvement, so future investigations must delve into these aspects to gain a more nuanced understanding of the findings.

Finally, the current study found that neighborhood factors (moderate-to-low opportunity score and greater distance from centers offering neurodevelopmental follow-up) were associated with a low rate of attendance. In this context, opportunity scores included indicators of income inequality, limited job opportunities, low high school graduation rates, and a lack of age diversity in the community where the family lived. Existing research has shown that income inequality and median income play a significant role in clinic no-show rates at academic pediatric otolaryngology practices.^34^ Similarly, another academic center in Michigan, U.S. also found that clinic no-shows, defined as missing three or more pediatric otolaryngology appointments, were more common in children from areas with lower income.^35^ Fraiman et al. conducted a retrospective study of 477 infants from a single large urban academic Level III neonatal ICU in Massachusetts, U.S. from January 2015 to June 2017. Mothers who lived in neighborhoods with a very low child opportunity index were less likely to attend post-discharge programs.^36^

Previous research has linked greater distance from the healthcare center to unsuccessful or missed follow-up appointments in various high-risk populations.^8,17,37–39^ Loccoh et al. identified 552 infants with CHD who underwent cardiac surgery and were discharged from C.S. Mott Children’s Hospital in Michigan, U.S.; one-tenth of the sample lived more than 200 miles from the center and had 2.86 higher odds of non-attendance at follow-up.^8^ Perez et al. analyzed clinic no-show rates across a variety of pediatric subspecialties at Lucile Packard Children’s Hospital Stanford in California, U.S. Traveling more than 50 miles was associated with 1.09 higher odds of no-show compared to shorter distances.^37^ Overall, the findings indicate the need for more satellite locations, referrals to local clinics, and potential home assessments to improve attendance for neurodevelopmental follow-up.

A lack of easy and accessible public transportation or transportation could be a major contributor to low attendance rates in neighborhoods with limited economic opportunities and a greater distance to the center for follow-up.^17,40,41^ A further plausible reason for low attendance is the low density of healthcare providers in low-income areas, as well as the long distances from metropolitan areas.^42,43^ Another consideration is the travel time between urban and rural areas, which may influence attendance rates. Nonetheless, our study did not distinguish between urban and rural areas, and further research is needed.

While interpreting findings, it is important to acknowledge the limitations of the current study. First and foremost, convenience sampling has the potential to introduce sampling bias. The current sample was primarily drawn from a large national patient and family advocacy organization, which could have resulted in a sample that was not completely representative. Given that many of our participants were part of a support and advocacy group with resources to help them learn about neurodevelopmental follow-up, attendance rates in other communities may be much lower than those found in this study. Another limitation was a lack of sociodemographic diversity in our sample, which was mostly made up of White or Caucasian populations. Moreover, this could potentially be due to the survey being available only in English. There is also the possibility that primary caregiver responses in this study were subject to recall bias; however, verification of the provided information with institutional records was not possible. Finally, the study sample lacks a comparison group, making it difficult to draw firm conclusions about the distinct impact of various factors on attendance for neurodevelopmental follow-up in children with CHD.

In conclusion, the current study underscores the necessity of targeted interventions, with a particular emphasis on the social determinants of health framework, to improve the attendance of children with CHD at neurodevelopmental follow-up. To improve their surveillance and follow-up, multifaceted innovative strategies addressing concerns at the healthcare system, healthcare professionals, and parental/primary caregiver levels must be prioritized. More formal follow-up programs for older children are required within the healthcare system. Establishing additional satellite locations may help to alleviate the travel burden for families with high-risk children. Furthermore, combining telehealth innovations with the development of instruments and questionnaires tailored specifically to children with CHD may allow for a better understanding of their developmental status during cardiac follow-up and/or primary care visits. Educational programs may benefit both healthcare professionals and caregivers. Given that many pediatric CHD cases are managed by their primary care pediatrician, it is critical to raise awareness among these providers about guidelines and the importance of neurodevelopmental follow-up care. Furthermore, increasing primary caregivers’ awareness of neurodevelopmental delays/deficits and available follow-up services can help them advocate for their children and better understand the risks involved. These strategies may increase attendance at neurodevelopmental follow-up appointments, allowing for early detection of impairments and targeted interventions, as well as continuity of care into adolescence and young adulthood, facilitating a successful transition to adult care programs.

## Data Availability

Datasets generated and/or analyzed for this study will be available on reasonable request from the corresponding author.
